# Differences in spinal structural lesions between patients with early axSpA and non-axSpA chronic back pain: 2-year SPACE cohort results

**DOI:** 10.1093/rheumatology/keaf500

**Published:** 2025-09-17

**Authors:** Gizem Ayan, Liese de Bruin, Miranda van Lunteren, Manouk de Hooge, Ana Bento da Silva, Mary Lucy Marques, Monique Reijnierse, Victoria Navarro-Compán, Marleen van de Sande, Inger Jorid Berg, Roberta Ramonda, Sofia Exarchou, Désirée van der Heijde, Floris van Gaalen, Sofia Ramiro

**Affiliations:** Department of Rheumatology, Leiden University Medical Center, Leiden, The Netherlands; Department Rheumatology, Ankara Research and Training Hospital, Ankara, Türkiye; Department of Rheumatology, Leiden University Medical Center, Leiden, The Netherlands; Department of Rheumatology, Leiden University Medical Center, Leiden, The Netherlands; Department of Rheumatology, Leiden University Medical Center, Leiden, The Netherlands; Department of Rheumatology, Ghent University Hospital, Ghent, Belgium; Department of Rheumatology, Leiden University Medical Center, Leiden, The Netherlands; Department of Rheumatology, Leiden University Medical Center, Leiden, The Netherlands; Rheumatology Department, Coimbra Local Health Unit, Coimbra, Portugal; Department of Radiology, Leiden University Medical Center, Leiden, The Netherlands; Department of Rheumatology, Hospital Universitario La Paz, IdiPaz, Madrid, Spain; The Department of Rheumatology and Clinical Immunology, Amsterdam University Medical Center, Amsterdam, The Netherlands; Center for Treatment of Rheumatic and Musculoskeletal Diseases (REMEDY), Diakonhjemmet Hospital, Oslo, Norway; Rheumatology Unit, Department of Medicine-DIMED, University of Padova, Padova, Italy; Department of Clinical Sciences Malmö, Rheumatology, Lund University, Malmö, Sweden; Department of Rheumatology, Leiden University Medical Center, Leiden, The Netherlands; Department of Rheumatology, Leiden University Medical Center, Leiden, The Netherlands; Department of Rheumatology, Leiden University Medical Center, Leiden, The Netherlands; Department of Rheumatology, Zuyderland Medical Center, Heerlen, The Netherlands

**Keywords:** axSpA, imaging, radiography, MRI, structural lesions

## Abstract

**Objectives:**

To compare spinal structural lesions on radiography and magnetic resonance imaging (MRI) over 2 years, between patients with early axial spondyloarthritis (axSpA) and non-axSpA chronic back pain.

**Methods:**

Patients from the SPACE cohort with available radiography or MRI at both baseline and 2years were included. Spinal lesions on radiography were assessed by the modified Stoke Ankylosing Spondylitis Spine Score (mSASSS), corner MRI lesions by the modified Canada–Denmark scoring system. Baseline spinal structural lesions and 2-year changes were compared between axSpA and non-axSpA. Generalized estimating equations were used to assess the change over 2 years, adjusting for age, sex, non-steroidal anti-inflammatory drug use and diagnosis.

**Results:**

Radiography data from 318 patients (67% axSpA), MRI data from 351 patients (69% axSpA) were included. At baseline, the mean (SD) mSASSS was 0.6 (1.1) for both axSpA and non-axSpA. Over 2 years, mSASSS progression was minimal (0.01 units/year) in both groups. On MRI, axSpA patients had a mean of 1.4 (2.9) total structural lesions compared with 0.7 (2) in non-axSpA at baseline (*P = *0.12). Significant 2-year increase in structural lesions [0.5 (1.8)] was mainly due to fat lesions [0.5 (1.6)] in axSpA. On MRI, fat lesions changed at a rate of 0.16 units/year in axSpA (*P = *0.002) and −0.02 units/year in non-axSpA (*P = *0.70).

**Conclusion:**

Over 2 years, spinal structural damage typical for axSpA progressed minimally on radiography in axSpA and non-axSpA. On MRI, axSpA showed a significant increase in fat lesions, while non-axSpA had no progression. Fat lesions may be important to assess spinal changes from early disease onwards.

Rheumatology key messages

- Radiographic spinal damage was minimal and similar between early axSpA and non-axSpA CBP.
- Fat lesions progressed more in axSpA versus non-axSpA, but only with baseline spinal inflammation.
- Erosions, bone spurs and ankylosis showed no progression in both axSpA and non-axSpA.

## Introduction

Axial spondyloarthritis (axSpA) is an inflammatory rheumatic and musculoskeletal disease, characterized by axial inflammation, which in turn can lead to structural damage of the axial skeleton [1, 2]. This damage significantly influences patient outcomes, either directly or indirectly [3]. Recognizing the importance of structural damage, it has been defined as one of the main domains in the Assessment of Spondyloarthritis International Society (ASAS)/Outcome Measures in Rheumatology (OMERACT) core domain set for axSpA [4]. Due to the slow progression of structural damage in axSpA, its measurement is challenging. The modified Stoke Ankylosing Spondylitis Spine Score (mSASSS) on radiography remains a widely used and validated instrument for the assessment of structural spinal damage and is the instrument recommended in the axSpA ASAS core outcome set [5]. However, radiography has low sensitivity to assess spinal damage, particularly in the early phases of the disease. On the other hand, magnetic resonance imaging (MRI) can detect erosions and fat lesions in addition to bone spurs; however, little is known about their change over time [6].

‘Disease progression’ has been identified in a large international patient survey as the most common fear of patients with axSpA, with the most common hope being ‘stop progression’ [7]. Understanding the development and progression of structural damage in axSpA remains a critical area of research. Studying the evolution of structural damage requires assessing a patient population over time, starting from the early stages of the disease [8]. However, focusing solely on the disease may not provide the complete picture, as other underlying processes, such as mechanical factors contributing to damage, could also play a role. Therefore, including a comparison group of patients with chronic back pain (CBP), without axSpA, has additional value. While several studies have examined structural damage in an inception cohort of axSpA using radiography or MRI, no previous study has compared structural damage and its progression between patients with and without axSpA within the same cohort [9–11]. Such comparisons are crucial to identify disease-specific changes and get a better understanding of the pathophysiology of axSpA. The SpondyloArthritis Caught Early (SPACE) cohort is unique as it is currently the only inception cohort of axSpA that also includes patients with non-axSpA CBP, with the same 2-year follow-up protocol for all participants regardless of diagnosis.

In this study, we aimed to investigate the difference in spinal structural lesions as assessed on radiography and MRI at baseline and 2-year follow-up between patients with early axSpA and non-axSpA CBP from the SPACE cohort. Additionally, we investigated the effect of baseline inflammation on the change of structural lesions over time.

## Methods

### Study population

Data from the SPACE cohort were used for this study. The cohort includes individuals over 16 years of age who presented with CBP lasting ≥3 months but ≤2 years and starting before the age of 45 years. Participants were recruited from outpatient clinics across 14 centres in the Netherlands, Norway, Italy and Sweden. Diagnostic assessments comprised a detailed patient history, physical examination, acute phase reactants measurement (C-reactive protein and erythrocyte sedimentation rate), HLA-B27-testing and imaging. Imaging included radiography and MRI of both sacroiliac joints (SIJ) and spine. Eligible patients (patients with at least one major specific SpA feature or two minor pre-specified and specific SpA features) underwent the same evaluations during follow-up visits. The details of this cohort have been described previously [12].

Rheumatologists rated their level of confidence in the diagnosis (axSpA or non-axSpA) on an 11-point numeric scale (low to high confidence) at each visit. After 2 years of follow-up, six diagnostic categories were defined: ‘Definite axSpA’, ‘Definite non-axSpA’, ‘Most likely axSpA’, ‘Most likely non-axSpA’, ‘Possible axSpA’ and ‘Possible non-axSpA’ [12]. For the current study, patients with definite or most likely axSpA/non-axSpA were included, category of patients with possible diagnosis was excluded and classified as axSpA/non-axSpA, respectively. Patients who had both baseline and 2-year imaging data of the spine, either on radiography or MRI, were included in the analysis.

The SPACE study protocol was approved by the Medical Ethics Committee of Leiden University Medical Centre (P08.105), the Regional Committee for Medical and Health Research Ethics in South-East Norway (2014/426), the Ethics Committee ‘Azienda Ospedaliera di Padova’ (2438P) and the Regional Research Ethics Committee in Lund, Sweden (2010/653). All participants gave written informed consent before the start of the study in accordance with the Declaration of Helsinki. The dataset was locked on 20 July 2023.

### Imaging and assessment

Radiographs and MRIs of the spine were performed at baseline, 1 year and 2 years. Radiographs were scored by three trained experts and MRIs were evaluated by two of the same readers. Readers were calibrated prior to the initial image assessments, and both modalities were scored independently. Readers were blinded to demographic and clinical data as well as the chronological order of the images.

The mSASSS was used for radiography assessments [13]. Individual missing vertebral corners were imputed based on change scores for each individual reader, following a previously described method [14, 15]. The total score for each patient ranged from 0 to 72 and was calculated as the average of the score of the three readers. Two-year change scores and the number of syndesmophytes were also computed, first at the patient level per reader and then as an average of the three readers. Lastly, the proportion of patients with any syndesmophyte was calculated using the agreement of the majority (≥2 out of 3) of the readers.

Structural lesions on spinal MRIs were assessed using a modified version of the Canada–Denmark (CANDEN) method focusing on presence/absence of corner lesions [6, 16]. In this method, 23 vertebral units (VUs) were divided into four quadrants. Erosions, fat lesions and bone spurs were scored per quadrant using a binary classification (yes/no), with abnormalities considered present, yielding a potential score range of 0–92. Ankylosis was scored per two quadrants and assessed based on its presence on at least one slice, with scores ranging from 0 to 46. The number of total structural lesions, including erosions, bone spurs, fat lesions and ankylosis was calculated, resulting in a score ranging from 0 to 322. Two-year change scores were also computed. Similar to the radiography parameters, continuous variables (total number of structural lesions, erosions, bone spurs, fat lesions, and ankylosis and their 2-year change scores) were calculated by averaging the scores of the two readers. For dichotomous variables (number of patients ≥3 erosions, ≥3 fat lesions, ≥5 fat lesions, ≥5 fat lesions and/or erosions, ≥1 bone spur), the relevant cut-off was considered met only if both readers agreed [17, 18]. In a sensitivity analysis, MRI structural lesions according to each individual reader were used.

Inflammatory lesions on spinal MRI were assessed with the total spine Spondyloarthritis Research Consortium of Canada (SPARCC) score (0–414), calculated as the average of two readers [19, 20]. Moreover, the modified New York criteria (mNY) and the ASAS classification for active sacroiliitis on MRI-SIJ were applied to define the presence and absence of sacroiliitis, as determined by majority reader agreement [21, 22].

### Statistical analysis

Interobserver reliability was evaluated by intraclass correlation coefficient (ICC) and the smallest detectable change (SDC). SDC represents the minimum change in an individual that can be assessed beyond measurement error calculated by the two different formulae according to the number of assessors. The formula for the SDC when there are three readers is: SDC=±1.96×(SEMchange-score/√k). SEMchange-score is the standard error (SE) of measurement of the change score which is equal to residual error derived from a two-way ANOVA and k is the number of readers. When there are two readers the formula is SDC = ±1.96 × [SDdiffchange-score/(√2 × √k)]. SDdiffchange-score is the standard deviation of the difference between the change scores of two readers [23]. Descriptive statistics were reported for both axSpA and non-axSpA patients. The Wilcoxon matched-pairs signed-rank test was performed to compare continuous variables between baseline and 2 years and the Wilcoxon rank-sum test was used for comparisons between the axSpA and non-axSpA groups. Dichotomous variables between baseline and 2 years were compared using the McNemar test, and comparisons between diagnostic groups were made using the χ^2^ test or Fisher’s exact test, as appropriate.

To assess the change of structural lesions of the spine over 2 years in patients with axSpA and non-axSpA we used generalized estimating equations models (GEE), with separate models for radiography (mSASSS) and MRI (erosions, fat lesions, bone spurs, ankylosis, total structural lesions). GEE is a method for analysing longitudinal data to describe the change of an outcome over time, making use of all available data (in this case baseline, 1-year and 2-year imaging scores) which is also robust to deviations from normality [24, 25]. Each structural lesion was modelled separately as the dependent variable, with time (in years) as the independent variable, whose regression coefficient indicated the yearly progression. Exchangeable correlation structure was used in all GEE models. The diagnostic group (axSpA *vs* non-axSpA) was first tested as an interaction term with time in years. If the interaction was statistically significant (*P*-value <0.15) and clinically relevant, results were stratified per diagnostic group, otherwise, diagnostic group was included as covariate. Adjustments were made for age at baseline, sex, use of non-steroidal anti-inflammatory drugs (NSAIDs; yes *vs* no). When a significant change over time was found for a structural lesion within the axSpA group, the impact of MRI spinal inflammation on this change was explored testing the interaction between time and total spine SPARCC score at baseline. If significant, the model was stratified based on the median total SPARCC score. If not, the model was adjusted for the total SPARCC score. The same strategy was also used to test the modifying effect of the presence of sacroiliitis at baseline (according to mNY or ASAS MRI criteria) within the axSpA group. Analyses were conducted in Stata SE V.18 (Statacorp, College Station, TX, USA).

## Results

Overall, the SPACE cohort included 702 patients, of whom 669 were eligible for this analysis (33 were excluded due to possible diagnosis). In total, 434 patients had both baseline and 2-year follow-up completed, of which 318 [axSpA, *n* = 214 (67%); non-axSpA, *n* = 104] had radiography, and 351 [axSpA, *n* = 242 (69%); non-axSpA, *n* = 109] had MRI at both time points (Supplementary Fig. S1).

The mean age and symptom duration were similar between axSpA and non-axSpA in both imaging groups. There were more males, higher frequency of HLA-B27 positivity and ASAS inflammatory back pain criteria positivity in axSpA patients (Table 1).

**Table 1.** Baseline characteristics of the patients included for analysis of radiography and magnetic resonance imaging assessments, stratified by diagnosis

		Radiography *n* = 318		Magnetic resonance imaging *n* = 351	
		AxSpA (*n* = 214)	Non-axSpA (*n* = 104)	AxSpA (*n* = 242)	Non-axSpA (*n* = 109)
Age (years)		29 (8)	31 (8)	30 (8)	31 (8)
Male gender		116 (54)	24 (23)	132 (55)	29 (27)
axSpA	Radiographic	12 (13)	NA	12 (12)	NA
	Non-radiographic	77 (87)	NA	88 (88)	NA
ASAS-MRI SIJ criteria positivity		85 (41)	4 (4)	99 (41)	4 (4)
Symptom duration (months)		13 (7)	14 (7)	13 (7)	13 (7)
HLA-B27 positivity		163 (76)	26 (25)	177 (73)	31 (28)
IBP ASAS criteria		151 (71)	62 (60)	170 (70)	69 (63)
ASDAS		2.4 (0.9)	2.6 (0.8)	2.4 (0.9)	2.7 (0.8)
ASDAS ≥ 2.1		123 (62)	68 (74)	140 (62)	74 (75)
Elevated CRP (≥5 mg/L)		73 (34)	23 (22)	80 (33)	22 (20)
NSAID use		157 (73)	67 (64)	178 (74)	74 (68)
bDMARD use^a^		4 (2)	1 0	7 (3)	1 0
Smoking status	Ever smoking status	91 (44)	49 (49)	93 (40)	53 (50)
	Current smoking status	36 (17)	20 (20)	40 (17)	22 (21)

Data presented as mean (SD) or *n* (%) as appropriate.

a Patients received bDMARDs at baseline because of concomitant diseases, e.g. psoriasis, inflammatory bowel disease or recurrent uveitis.

ASAS: Assessment of Spondyloarthritis International Society; ASDAS: Axial Spondyloarthritis Disease Activity Score; axSpA: axial spondyloarthritis; bDMARD: biologic disease modifying anti-rheumatic drug; CRP: C-reactive protein; HLA-B27: human leucocyte antigen-B27; IBP: inflammatory back pain; MRI: magnetic resonance imaging; NA: not applicable; NSAID: non-steroidal anti-inflammatory drug; SIJ: sacroiliac joint.

### Radiography

**Figure 1.**
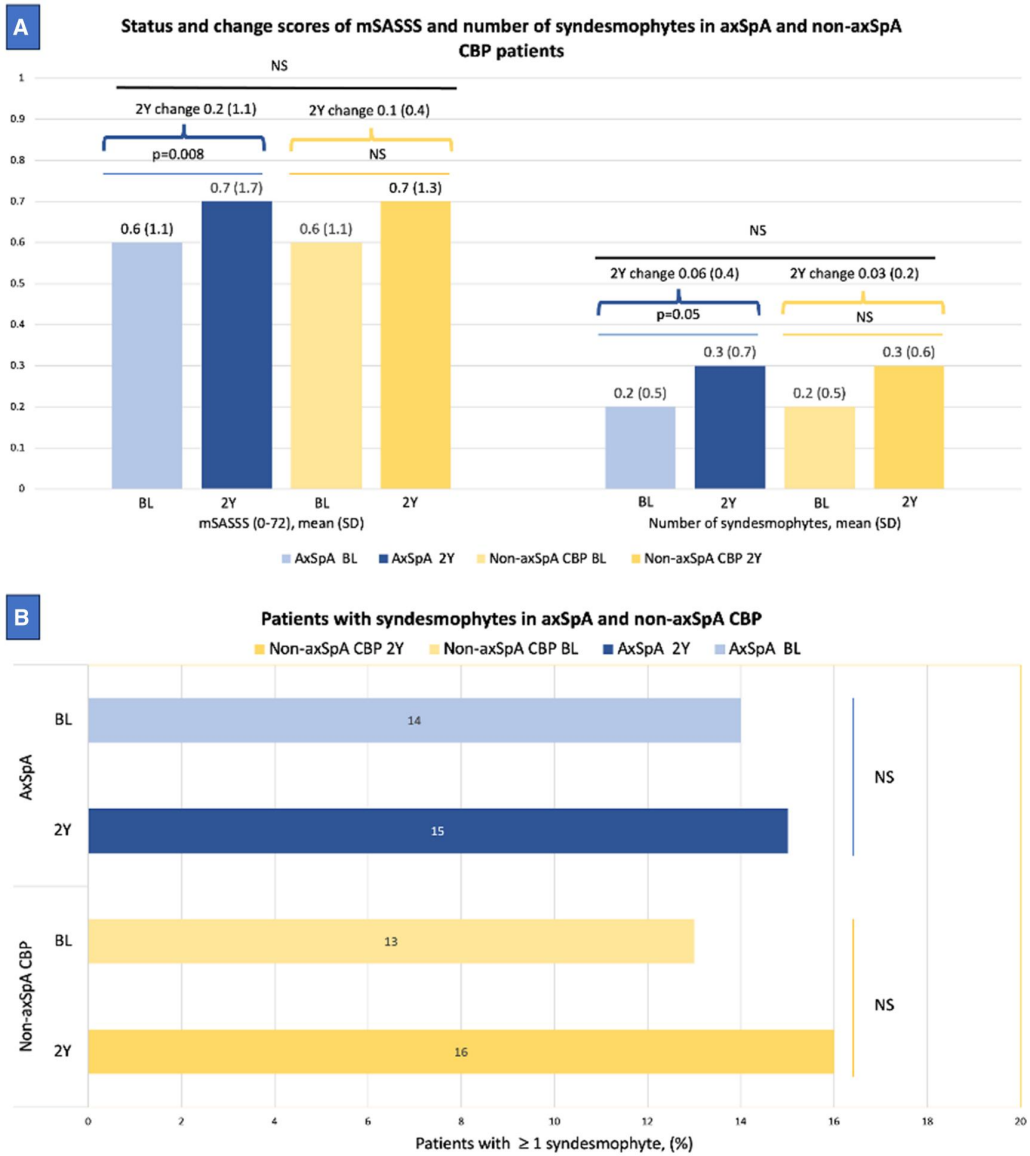
mSASSS and syndesmophytes in axSpA and non-axSpA CBP. (**A**) Status and change scores of mSASSS and number of syndesmophytes in axSpA and non-axSpA CBP patients on radiography. (**A**) displays the mean (SD) modified Stoke Ankylosing Spondylitis Spine Score (mSASSS) and the mean (SD) number of syndesmophytes in axSpA at baseline and at 2 years, as well as in non-axSpA at baseline and at 2 years using bar charts. The lines connecting the bars represent the *P*-values for the variables being compared (differences from baseline to 2 years in axSpA and non-axSpA groups). Above these *P*-values, the mean (SD) 2-year change scores are shown for the respective groups of patients, connected by curly brackets. The line above the 2-year change scores indicates the *P*-value for the difference in the change scores between the axSpA and non-axSpA groups. (**B**) Patients with syndesmophytes in axSpA and non-axSpA on radiography. (**B**) illustrates the percentage of patients with at least one syndesmophyte in axSpA at baseline and at 2 years, as well as in non-axSpA at baseline and at 2 years. The perpendicular lines connecting the bars represent the *P*-values for the variables being compared (differences from baseline to 2 years in axSpA and non-axSpA groups). No differences between the axSpA and non-axSpA groups at baseline or at 2-year assessments for individual lesions in (A) and (B) was observed. 2Y: 2-year; BL: baseline; CBP: chronic back pain; NS: non-significant

The SDC over 2 years in mSASSS was 0.70 and ICC values were 0.79 (0.76–0.83) and 0.85 (0.82–0.88) for baseline and 2-year assessments, respectively and 0.63 (0.57–0.68) for the 2-year change. The mean (SD) mSASSS at baseline was 0.6 (1.1) for both the axSpA and non-axSpA groups. At 2 years, the mean mSASSS was 0.7 (1.7) for axSpA and 0.7 (1.3) for non-axSpA, and no statistically significant difference was observed between the two groups at each time point. A statistically significant, though numerically minimal, increase in mSASSS was detected within the axSpA group from baseline through 2 years. The difference was not significant in the non-axSpA group. Additionally, there was no significant difference between the diagnostic groups in the change score (0.2 mSASSS units in axSpA and 0.1 mSASSS units in non-axSpA) (Fig. 1A).

The mean (SD) number of syndesmophytes was 0.2 (0.5) at baseline in both groups and 0.3 (0.7) in axSpA and 0.3 (0.6) in non-axSpA groups at 2 years. The axSpA group showed a small but significant increase in the number of syndesmophytes from baseline to 2 years. There was a minimal increase in the non-axSpA group, and no significant difference was found when both timepoints were compared. Moreover, there was no significant difference between axSpA and non-axSpA groups at either baseline or at 2 years. Similarly, 2-year change in syndesmophytes was comparable between axSpA and non-axSpA patients (Fig. 1A). In the number of patients with at least one syndesmophyte, axSpA and non-axSpA showed similar results (Fig. 1B).

**Figure 2.**
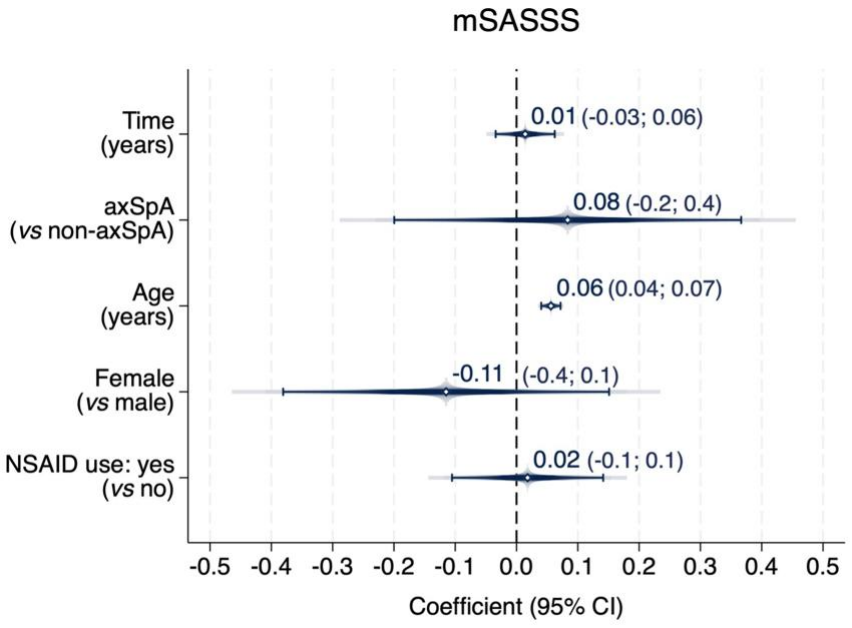
Progression in mSASSS over 2 years (multivariable model). Two-year progression in the modified Stoke Ankylosing Spondylitis Spine Score (mSASSS) using a multivariable generalized estimating equations (GEE) model. The y-axis represents the variables: time (in years) as the independent variable, diagnosis (axSpA *vs* non-axSpA), age (in years), sex (female *vs* male) and non-steroidal anti-inflammatory drug (NSAID) use (yes *vs* no). The x-axis shows the coefficients with their corresponding 95% confidence intervals

The course of mSASSS over the 2-year follow-up, assessed using the GEE model, showed no significant interaction between diagnosis (axSpA *vs* non-axSpA) and time, indicating that the progression of mSASSS over time was comparable between groups. Adjusted for confounders, there was no mSASSS progression in the total population, more specifically 0.01 units per year (Fig. 2).

### MRI

**Figure 3.**
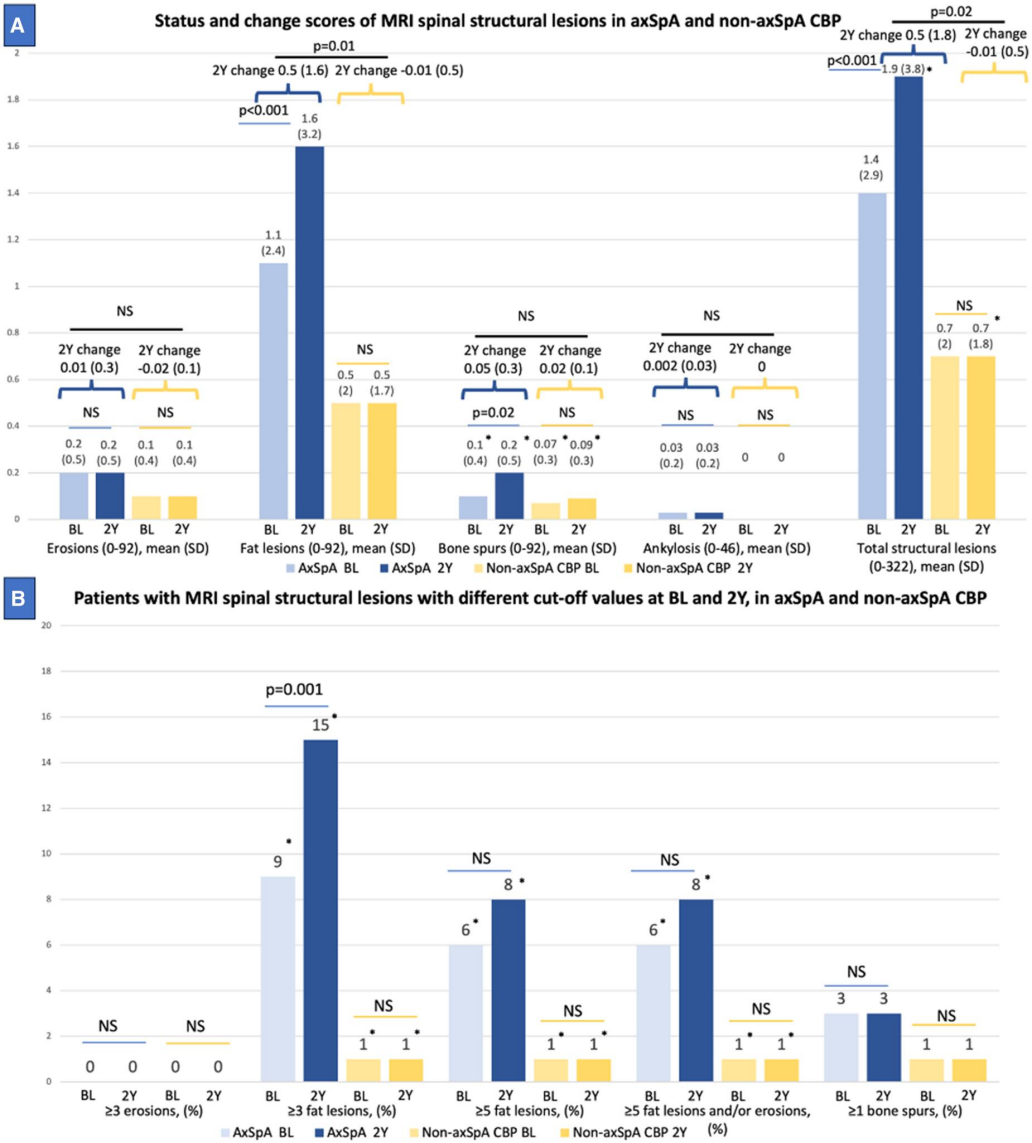
MRI spinal structural lesions in axSpA and non-axSpA CBP. (**A**) Status and change scores of MRI spinal structural lesions in axSpA and non-axSpA CBP. (**A**) presents the mean (SD) number of erosions, fat lesions, bone spurs, ankylosis and total structural lesions in axSpA at baseline and at 2 years, as well as in non-axSpA at baseline and at 2 years, using bar charts. The lines connecting the bars represent the *P*-values comparing changes from baseline to 2 years within the axSpA and non-axSpA groups. Above these *P*-values, the mean (SD) change scores over 2 years are displayed for each group, connected by curly brackets. The line above the 2-year change scores indicates the *P*-value for the difference between the axSpA and non-axSpA groups. Statistically significant differences between the axSpA and non-axSpA groups at baseline or at 2-year assessments for individual lesions are indicated by an asterisk (*). (**B**) Patients with MRI spinal structural lesions at baseline, 2 years in axSpA and non-axSpA. (**B**) shows the percentage of patients with ≥3 erosions, ≥3 fat lesions, ≥5 fat lesions, ≥5 fat lesions and/or erosions and ≥1 bone spur in axSpA at baseline and at 2 years, as well as in non-axSpA at baseline and at 2 years, using bar charts. The lines connecting the bars represent the *P*-values comparing changes from baseline to 2 years within the axSpA and non-axSpA groups. Statistically significant differences between the axSpA and non-axSpA groups at baseline or at 2-year assessments for each dichotomous variable are indicated by an asterisk (*). 2Y: 2-year; BL: baseline; CBP: chronic back pain; NS: non-significant

The SDC over 2 years was 0.4 for the number of erosions, 1.5 for the number of fat lesions, 0.5 for the number of bone spurs, 0.05 for the number of ankylosis and 3.1 for the number of total structural lesions. ICC value for total structural lesions was 0.81 (0.76–0.84) for both baseline and 2-year assessments and 0.58 (0.48–0.66) for 2-year change. The mean number of fat lesions was 1.1 (2.4) in axSpA and 0.5 (2.0) in non-axSpA (*P = *0.19) at baseline. At 2 years, the mean number of fat lesions was 1.6 (3.2) in axSpA and 0.5 (1.7) in non-axSpA group (*P = *0.06). The mean number of fat lesions was significantly higher at 2 years compared with baseline in the axSpA group (*P* <0.001) while no significant difference was observed in the non-axSpA group. In terms of change scores, the axSpA group had a higher 2-year increase in the number of fat lesions compared with the non-axSpA group [0.5 (1.6) *vs* −0.01 (0.5), *P = *0.01]. No notable changes were observed in the number of erosions, bone spurs or ankylosis between baseline and 2 years in either the axSpA or non-axSpA groups. The number of total structural lesions mirrored the fat lesions pattern, increasing only in the axSpA group [from 1.4 (2.9) – 1.9 (3.8) (*P* <0.001)] with a higher mean 2-year change score [0.5 (1.8) in axSpA *vs* −0.01 (0.5) in non-axSpA, *P = *0.02] (Fig. 3A).

At baseline, the number of patients with ≥3 fat lesions was 21 (9%) in axSpA and 1 (1%) in non-axSpA (*P = *0.004). At 2 years, the number of patients with ≥3 fat lesions were 35 (15%) *vs* 1(1%) in axSpA and non-axSpA, respectively (*P* <0.001). The difference between baseline and 2 years for the number of patients with ≥3 fat lesions was significant in the axSpA group (*P = *0.001). No significant differences were noted within the non-axSpA group between baseline and 2 years. A significant difference between the axSpA and non-axSpA groups was also found for the ≥5 fat lesions cut-off as well as ≥5 fat lesions and/or erosions at both baseline and at 2 years (Fig. 3B).

The sensitivity analysis yielded results that were generally consistent with the main analysis. Reader 2 identified some more differences at 2 years compared with baseline and compared with Reader 1 (Supplementary Tables S1 and S2).

**Figure 4.**
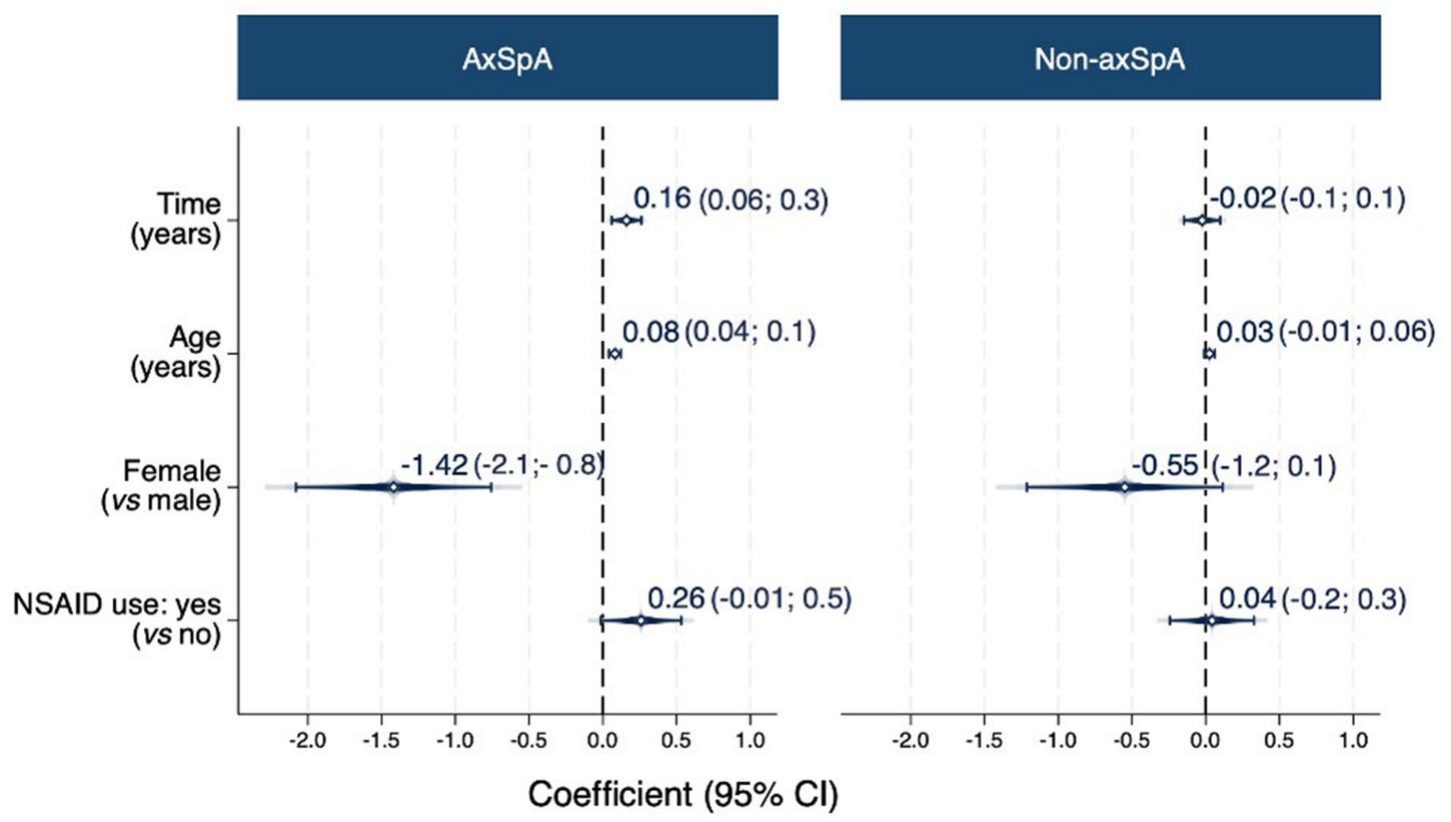
Progression in the number of fat lesions over 2 years (multivariable model). Two-year progression in the number of fat lesions using a multivariable generalized estimating equations (GEE) model in both axSpA and non-axSpA groups. The y-axis represents the variables: time (in years) as the independent variable, age (in years), sex (female *vs* male) and non-steroidal anti-inflammatory drug (NSAID) use (yes *vs* no) as adjusted variables. The x-axis shows the coefficients with their corresponding 95% confidence intervals

**Figure 5.**
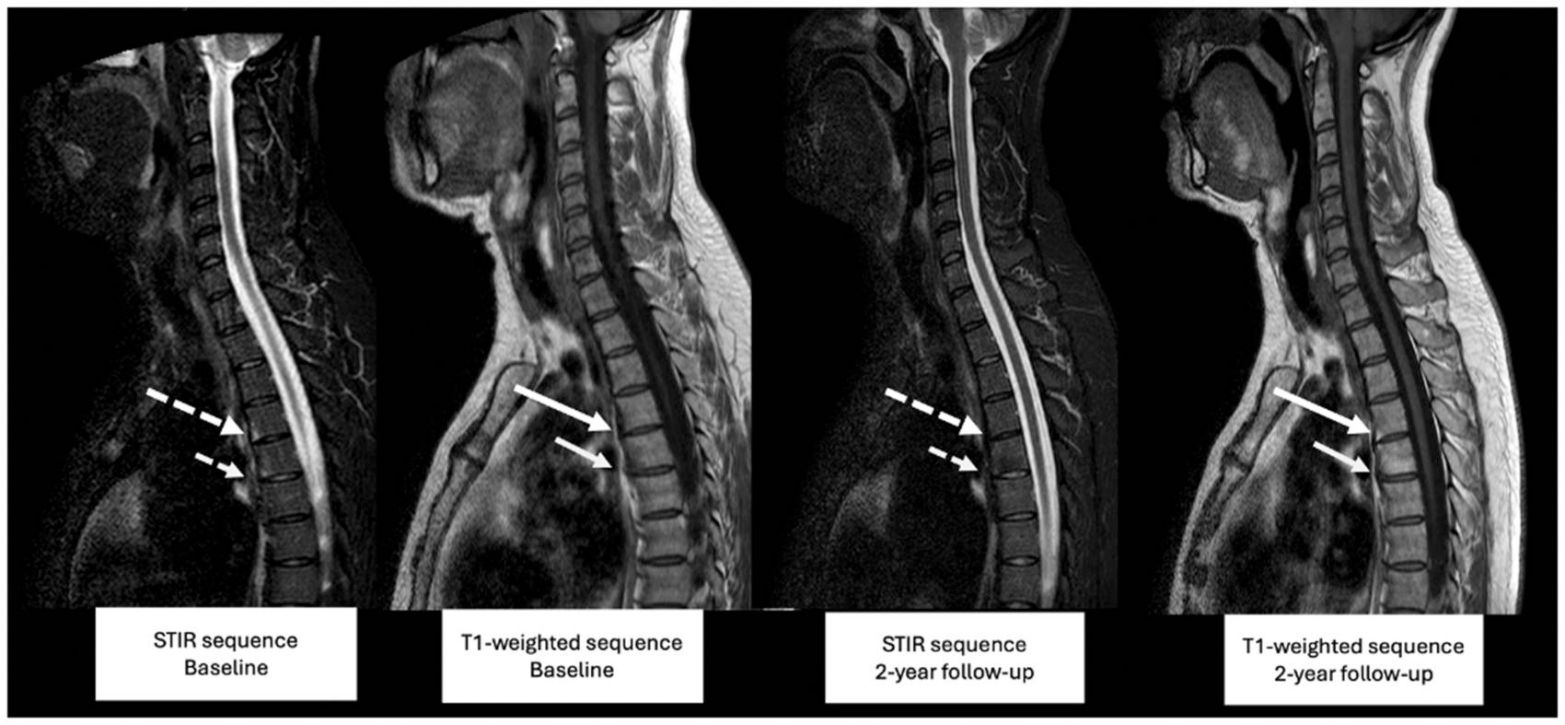
MRI lesion progression in axSpA: from bone marrow oedema to fat lesion or no change. Bone marrow oedema (BME) in the lower anterior quadrant of T4 at baseline (large, dashed arrow, STIR) without a corresponding structural lesion on T1-weighted imaging (large, solid arrow) progressed to a fat lesion after 2 years (large, solid arrow, T1-weighted), without corresponding inflammation (large, dashed arrow, STIR, 2-year). BME in the lower anterior quadrant of T5 at baseline (small, dashed arrow, STIR) without a corresponding structural lesion (small, solid arrow, T1-weighted) showed no new fat lesion after 2 years (small, solid arrow, T1-weighted) and no inflammation (small, dashed arrow, STIR, 2-year). Lesions were present on more than one consecutive slice

The GEE model on fat lesions revealed an interaction (*P = *0.006) between time and diagnosis (axSpA *vs* non-axSpA), which led to a stratification into both groups. The number of fat lesions changed at 0.16 units/year in axSpA patients, whereas non-axSpA patients exhibited no change, namely −0.02 units/year (Fig. 4). Moreover, in the axSpA group fewer fat lesions were observed in females. Following this, impact of spinal inflammation on fat lesions was tested, and an interaction between SPARCC at baseline and time in years was shown in the axSpA group (*P* <0.001) but not in the non-axSpA group (*P = *0.34). Subsequently, in the axSpA group, the model was stratified according to the median of the baseline SPARCC (i.e., 0). In the group without spinal inflammation (SPARCC of 0, *n* = 133), no change in fat lesions was found (−0.02 units/year); in the group with spinal inflammation (SPARCC > 0, *n* = 109), the yearly change of fat lesions was 0.4 units/year. Fig. 5 presents an example of an axSpA case with and without progression of fat lesions, together with the corresponding baseline images demonstrating inflammation at the same anatomical sites. In the axSpA group, the interaction between time and baseline mNY (positive, *n* = 12) and ASAS MRI-SIJ (positive, *n* = 99) were not significant (*P = *0.46 and *P = *0.72, respectively) for fat lesions on modified CANDEN scoring. No change was observed in the number of erosions (0.001 units/year), bone spurs (0.01 units/year) or ankylosis (−0.001 units/year).

## Discussion

This is the first study to directly compare spinal damage progression between early axSpA and non-axSpA CBP. In patients with early axSpA, fat lesions increased more in patients with axSpA compared with those with non-axSpA, but exclusively in patients with spinal inflammation at baseline. Other structural damage assessed on MRI, including erosions, bone spurs and ankylosis remained very low, without any change in both groups. Similarly, radiographic spinal damage was minimal, with negligible progression over time, and was not significantly different from that observed in non-axSpA CBP.

The 2-year mSASSS progression reported in our group with axSpA aligns with the 2-year progression of 0.2 mSASSS units reported in the Devenir des Spondyloarthropathies Indifférenciées Récentes (DESIR) cohort, another axSpA inception cohort [9]. Additionally, a comparative analysis of the DESIR and ILOS (a French multicentric cohort of patients with non-axSpA mechanical CBP) cohorts, representing axSpA and non-axSpA patients, respectively, similarly found no differences between baseline mSASSS scores or presence of syndesmophytes [11]. However, this study examined separate populations, making the comparison less robust. In contrast, our analysis included patients with CBP of unknown origin in a single cohort, followed prospectively using the same methodology. The categorization was made based on the final diagnosis at 2 years, allowing for more certainty in the diagnosis and a direct and accurate comparison between the groups.

Clinically, the presence of a syndesmophyte is often interpreted as indicative of axSpA. However, here we report patients with non-axSpA who have been considered as having syndesmophytes by blinded scoring within the limitation of mSASSS not being validated in the non-axSpA population. This reiterates that syndesmophytes on radiography are insufficient for a definite diagnosis of axSpA and underscore the presence of lesions in non-axSpA CBP that mimic syndesmophytes. This raises fundamental questions about the nature of these findings: Are they truly syndesmophytes, or could they represent measurement error? Alternatively, should these lesions be classified as ‘syndesmophyte-like’ structures instead? Such a classification would require further characterization, including analysis of their distribution, size and other defining features. An imaging modality like low-dose computed tomography (ldCT) could offer clearer insights into these structures compared with radiography.

There are several possible reasons for which structural progression appears to be low and, in fact, not even significantly different from that observed in non-axSpA CBP. There is less likelihood for the development of structural damage earlier in the disease process [1]. Moreover, although methodological limitations prevent an ultimate confirmation, indirect evidence suggests bDMARDs may slow structural damage progression by suppressing the inflammation [26]. Additionally, radiographic assessment is known to have low sensitivity to detect early changes. For instance, the CT Syndesmophyte Score (CTSS), assessed on ldCT is more sensitive to change than the mSASSS [27, 28]. Thus, more sensitive measurement methods could enable better investigation of spinal damage in early axSpA cohorts.

On MRI, the only difference observed between axSpA and non-axSpA is the change in fat lesions, seen as repair after inflammation. The change in fat lesions was shown to not be modified by the presence of sacroiliitis, though the groups were small. Our findings, which highlight differences only in the change of fat lesions between axSpA and non-axSpA CBP, hold significance from multiple perspectives. Fat lesions, considered as present above a certain threshold (from *≥*1 to *≥*6), have been shown to be specific to axSpA [18, 29–32]. This raises a key question: Do fat lesions have prognostic value in early axSpA patients? A biopsy study comparing patients with radiographic axSpA and those with degenerative disc disease (DDD) revealed that fat lesions in axSpA contain more adipocytes, while DDD is richer in inflammatory bone marrow. Immunofluorescence revealed higher osteoblast and minimal osteoclast activity in adipocyte-rich areas. This finding hypothetically suggests that early inflammation maintains a balance between osteoblast and osteoclast activity, which shifts towards osteoblast activity contributing to new bone formation as fat lesions increase [33]. This hypothesis aligns prior findings linking fat deposition in vertebral corners, regardless of inflammation, more strongly to radiographic progression than inflammation alone [34]. This finding is further supported by a ldCT study, which showed that vertebral corner inflammation and fat change were positively associated with syndesmophyte development at the same site after 2 years [35]. However, in both studies, ∼50% of new bone formed at corners without these lesions, supporting that other pathways also contribute to structural progression [34–36]. Additionally, previous literature showed in two independent cohorts that the majority of the effect of inflammation on syndesmophyte formation is not mediated by fat lesions [37]. However, these analyses were all conducted in patients with established disease and a long symptom duration. Whether changes in fat lesions in early disease genuinely reflect resolution of inflammation or contribute to structural damage remains an open question. Ideally, an inception cohort is better suited to clarify this relationship, especially as a previous study in the DESIR inception cohort has demonstrated an association between bone marrow oedema and subsequent fat lesions [38]. Nevertheless, in our current study, the absence of observable damage change limits our ability to draw conclusions about the prognostic significance of these lesions. Furthermore, our analyses were conducted at the patient level, and we did not assess the relationship between bone marrow oedema and subsequent fat lesions at the individual lesion level. Such lesion-level analyses could provide more granular insights into the evolution of lesions and may be an important focus for future research.

This study has a strength as the first study showing differences in structural lesions between axSpA and non-axSpA patients on both radiography and MRI using the same cohort followed by the same procedures. Notwithstanding, some limitations should be acknowledged. First, the non-axSpA group included in our cohort may not be fully representative of the general population with CBP, as all patients met the cohort entry criteria of having at least one major or two minor SpA features. Nevertheless, this reflects a subset of patients commonly referred to rheumatologists, making the findings more relevant for clinical practice and potentially providing better guidance for rheumatologists. In addition, our assessment covered only the anterior aspect of the cervical and lumbar spine on radiographs and corner lesions on MRI, a portion where typical spinal structural changes in axSpA are seen. Therefore, existing scores used to measure structural damage of the spine (e.g. CANDEN) focus on the anterior part of the spine and we have used them. However, we did not consider posterior element involvement and, therefore, our findings should be interpreted within this limitation. In our assessment, the SDC values were larger than the observed mean change; however, it should be emphasized that the SDC reflects the smallest detectable change at the individual patient level, rather than the mean change within a population. Another limitation is that we used only two readers in the MRI assessments. However, the sensitivity analysis on both readers separately confirmed the general findings. The CANDEN method used in MRI assessments is a modified version taking only the presence of corner lesions into consideration; however, this methodology has been used previously in other studies [10, 11, 38]. Moreover, the interaction analyses on the effect of a third variable (e.g. inflammation) on the change of structural lesions over time are limited due to their lack of statistical power and therefore considered exploratory.

In conclusion, our results demonstrated minimal progression in spinal structural lesions on radiography in both axSpA and non-axSpA. MRI revealed a significant 2-year increase in the number of fat lesions in the axSpA group, not seen in non-axSpA CBP. These findings suggest that, while radiography does not capture early structural progression, fat lesions assessed on MRI can potentially provide additional insights into disease evolution already in early phases of the disease. Our study underscores the need for further investigation into whether the difference in fat lesions progression has any prognostic significance in patients with early axSpA.

## Supplementary material

Supplementary material is available at *Rheumatology* online.

## Supplementary Material

keaf500_Supplementary_Data

## Data Availability

All data relevant to the study are included in the article or uploaded as online supplementary information.
