# Supplementary material for: Differences in spinal structural lesions between patients with early axSpA and non-axSpA chronic back pain: 2-year SPACE cohort results
Source: Rheumatology (Oxford). 2025 Sep 17;65(1):keaf500. doi: 10.1093/rheumatology/keaf500 (PMC12862369; doi:10.1093/rheumatology/keaf500)
Supplement: keaf500_Supplementary_Data [file keaf500_supplementary_data.docx]

**SUPPLEMENTARY FILE**

**Supplementary Figure S1: Flowchart of the patient selection process**

**
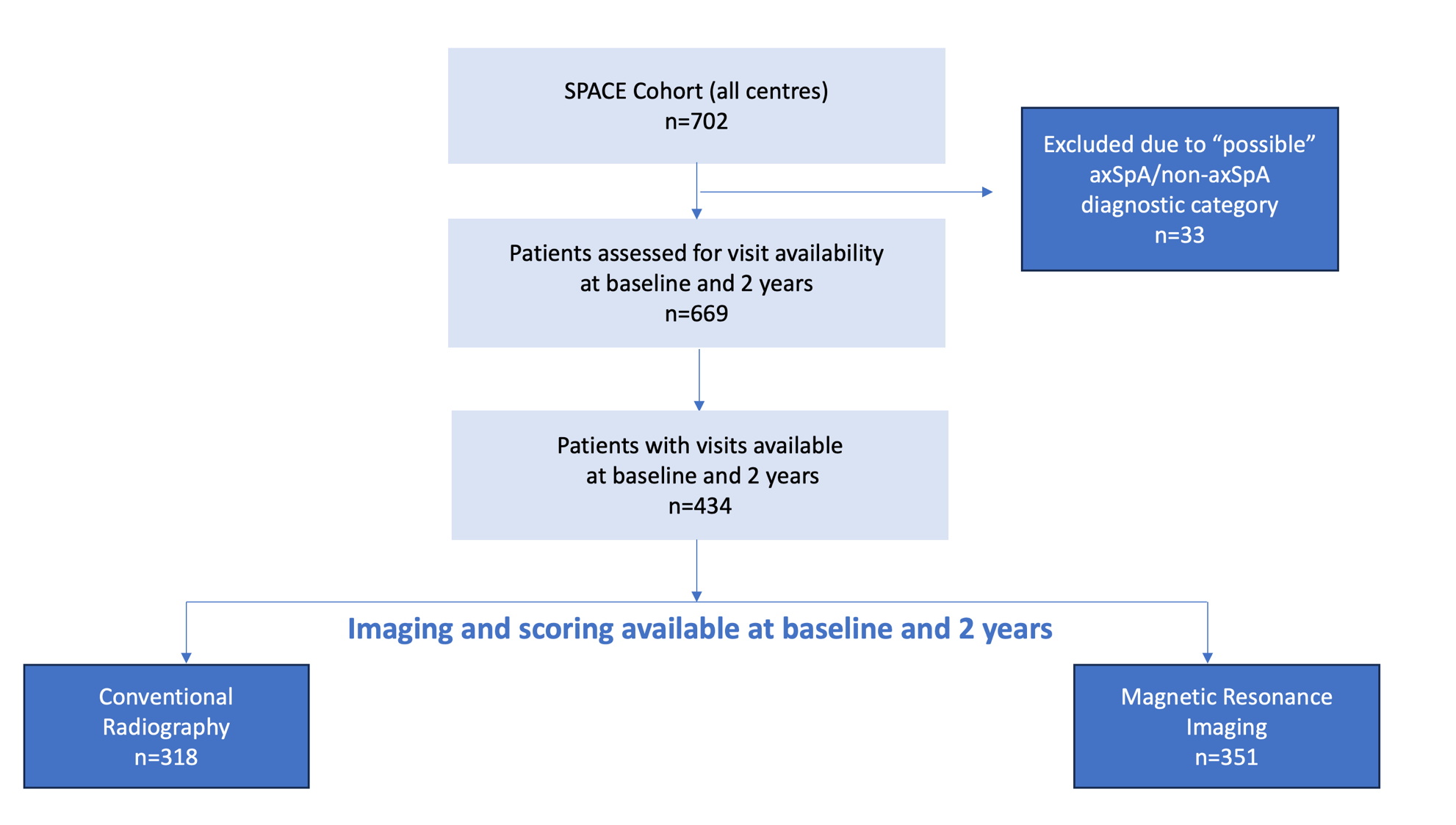
**

**Supplementary Table S1. Sensitivity analysis: status and change scores in axSpA and non-axSpA patients according to Reader 1**

|  | **Patients with axSpA, n=242*** | | | **Patients with non-axSpA, n=109*** | | | **P-value for BL axSpA**  **vs**  **non-axSpA** | **P-value for 2Y axSpA**  **vs**  **non-axSpA** |
| --- | --- | --- | --- | --- | --- | --- | --- | --- |
|  | **BL** | **2Y** | **p-value** | **BL** | **2Y** | **p-value** |  |  |
| Status scores |  |  |  |  |  |  |  |  |
| Erosions (0-92) | 0.1 (0.7) | 0.2 (0.7) | 0.08 | 0.1 (0.3) | 0.1 (0.3) | 1.00 | 0.43 | 0.80 |
| Fat lesions (0-92) | 0.8 (2.1) | 1.3 (3) | **<0.001** | 0.4 (2.3) | 0.4 (1.8) | 0.77 | 0.11 | **0.02** |
| Bone spurs (0-92) | 0.06 (0.3) | 0.1 (0.5) | 0.09 | 0.06 (0.4) | 0.08 (0.4) | 0.50 | 0.88 | 0.65 |
| Ankylosis (0-46) | 0.02 (0.2) | 0.03 (0.2) | 1.00 | 0 | 0 | 1.00 | 0.45 | 0.45 |
| Total structural lesions (0-322) | 1 (2.6) | 1.6 (3.6) | **<0.001** | 0.6 (2.4) | 0.5 (1.9) | 0.74 | 0.48 | 0.07 |
| ≥3 erosions | 2 (1) | 4 (2) | 0.5 | 0 | 0 | 1.00 | 1.00 | 0.32 |
| ≥3 fat lesions | 24 (10) | 42 (17) | **<0.001** | 1 (1) | 1 (1) | 1.00 | **0.001** | **<0.001** |
| ≥5 fat lesions | 17 (7) | 25 (10) | **0.02** | 1 (1) | 1 (1) | 1.00 | **0.02** | **0.001** |
| ≥5 fat lesions and/or erosions | 19 (8) | 26 (11) | 0.06 | 1 (1) | 1 (1) | 1.00 | **0.01** | **0.001** |
| ≥1 bone spurs | 10 (4) | 16 (7) | **0.03** | 4 (4) | 6 (6) | 0.5 | 1.00 | 0.81 |
| Change scores | **BL-2Y** | |  | **BL-2Y** | |  | **P-value** | |
| Change in erosions | 0.05 (0.4) | |  | -0.01 (0.1) | |  | 0.06 | |
| Change in fat lesions | 0.5 (1.9) | |  | -0.02 (0.6) | |  | **0.01** | |
| Change in bone spurs | 0.05 (0.5) | |  | 0.02 (0.1) | |  | 0.79 | |
| Change in ankylosis | 0.004 (0.1) | |  | 0 | |  | 1.00 | |
| Change in total structural lesions | 0.6 (2.2) | |  | -0.01 (0.7) | |  | **0.006** | |
| axSpA, axial spondyloarthritis; BL, at baseline; 2Y, at 2-year follow-up  *Data are presented as mean (SD) or n (%), as appropriate | | | | | | | | |

**Supplementary Table S2. Sensitivity analysis: status and change scores in axSpA and non-axSpA patients according to Reader 2**

|  | **Patients with axSpA, n=242*** | | | **Patients with non-axSpA, n=109*** | | | **P-value for BL axSpA**  **vs**  **non-axSpA** | **P-value for 2Y axSpA**  **vs**  **non-axSpA** |
| --- | --- | --- | --- | --- | --- | --- | --- | --- |
|  | **BL** | **2Y** | **p-value** | **BL** | **2Y** | **p-value** |  |  |
| Status scores |  |  |  |  |  |  |  |  |
| Erosions (0-92) | 0.2 (0.6) | 0.2 (0.6) | 0.31 | 0.2 (0.7) | 0.1 (0.6) | 0.50 | 0.18 | 0.13 |
| Fat lesions (0-92) | 1.4 (3) | 1.8 (3.8) | **<0.001** | 0.7 (1.8) | 0.7 (1.8) | 0.80 | 0.14 | **0.02** |
| Bone spurs (0-92) | 0.2 (0.7) | 0.3 (0.8) | 0.08 | 0.07 (0.4) | 0.09 (0.4) | 0.75 | **0.008** | **0.009** |
| Ankylosis (0-46) | 0.03 (0.3) | 0.03 (0.3) | 1.00 | 0 | 0 | 1.00 | 0.45 | 0.45 |
| Total structural lesions (0-322) | 1.9 (3.7) | 2.3 (4.5) | **<0.001** | 1 (2) | 1 (2) | 0.55 | 0.05 | **0.007** |
| ≥3 erosions | 5 (2) | 4 (1.7) | 1.00 | 1 (1) | 1 (1) | 1.00 | 0.67 | 1.00 |
| ≥3 fat lesions | 40 (17) | 50 (21) | **0.03** | 8 (7) | 7 (6) | 1.00 | **0.02** | **0.001** |
| ≥5 fat lesions | 22 (9) | 31 (13) | **0.02** | 4 (4) | 5 (5) | 1.00 | 0.08 | **0.02** |
| ≥5 fat lesions and/or erosions | 23 (10) | 33 (14) | **0.01** | 5 (5) | 6 (6) | 1.00 | 0.14 | **0.03** |
| ≥1 bone spurs | 36 (15) | 39 (16) | 0.5 | 6 (6) | 7 (6) | 1.00 | **0.01** | **0.02** |
| Change scores | **BL-2Y** | |  | **BL-2Y** | |  | **P-value** | |
| Change in erosions | -0.02 (0.3) | |  | -0.03(0.3) | |  | 0.99 | |
| Change in fat lesions | 0.5 (1.7) | |  | -0.01 (0.8) | |  | **0.009** | |
| Change in bone spurs | 0.04 (0.4) | |  | 0.02 (0.2) | |  | 0.40 | |
| Change in ankylosis | 0 | |  | 0 | |  | 1.00 | |
| Change in total structural lesions | 0.5 (1.8) | |  | -0.02 (0.8) | |  | **0.04** | |
| axSpA, axial spondyloarthritis; BL, at baseline; 2Y, at 2-year follow-up  *Data are presented as mean (SD) or n (%), as appropriate | | | | | | | | |
